# The Progress of T Cell Immunity Related to Prognosis in Gastric Cancer

**DOI:** 10.1155/2018/3201940

**Published:** 2018-02-27

**Authors:** Ming Wei, Duo Shen, Sachin Mulmi Shrestha, Juan Liu, Junyi Zhang, Ying Yin

**Affiliations:** ^1^Gastroenterology Department, Affiliated Zhongda Hospital of Southeast University, Nanjing, China; ^2^Department of Critical Care Medicine, Affiliated Zhongda Hospital of Southeast University, Nanjing, China

## Abstract

Gastric cancer is the fifth most common malignancy all over the world, and the factors that can affect progress and prognosis of the gastric cancer patients are various, such as TNM stages, invasive depth, and lymph node metastasis ratio. T cell immunity is important component of human immunity system and immunity responding to tumor and dysfunction or imbalance of T cell immunity will lead to serious outcomes for body. T cell immunity includes many different types of cells, CD4+ T cell, CD8+ T cell, memory cell, and so on, and each of them has special function on antitumor response or tumor immune escape which is revealed in lung cancer, colorectal cancer, breast cancer, ovarian cancer, and so on. But its correlation with gastric cancer is not clear. Our review was preformed to explore the relationship between the progress and prognosis of gastric cancer (GC) and T cell immunity. According to recent researches, T cell immunity may have an important role in the progress and prognosis of GCs, but its function is affected by location, category, related molecule, and interaction between the cells, and some effects still are controversial. More researches are needed to clarify this correlation.

## 1. Introduction

Gastric cancer is the fifth most common malignancy all over the world after lung, breast, colorectal cancers, and prostate. More than 70% of gastric cancer (677,000 cases) happened at developing countries (456,000 in men, 221,000 in women), and half the total located in Eastern Asia, especially in China [1]. Although the lifestyle and smoking play an important factor, the main risk factor for advanced gastric cancer is infection with the bacterium* Helicobacter pylori* [2]; T cell immunity is a hot topic in recent studies. During the development of cancer, T cells progressively dysfunction and exhaust; however the T cell responses are necessary to control tumors [3]. And they play important roles in several types of cancers like lung cancer [4], colorectal cancer [5], breast cancer [6], and ovarian cancer [7], but the relationship between the T cell immunity and progression and prognosis of GCs is not clear. And there are many subsets of T cells which play different roles in gastric cancer, CD4+ T cell, including regulatory T cells, CD8+ T cell, and CD45RO+ memory T cells [8]. The recent researches are more focused on regulatory T cells.

## 2. Subsets of T Cell and Molecules Related to Prognosis of Gastric Cancer

T cell immunity is important in tumor response, and there are many subsets of T cells which played different roles in gastric cancer, CD4+ T cell, including regulatory T cells, CD8+ T cell, CD45RO+ memory T cells, and other molecules related to T cell immunity.

### 2.1. CD4+ T and CD8+ T Lymphocytes

CD4+ T and CD8+ T are two important types of cells in T cell immunity.

CD4+ regulatory T cell is a major cell in self-tolerance and suppresses antitumor immunity [9]. CD4 T cells have effector functions by secreting multiple cytokines or activating other immune cells acting on immunity of tumor [3]. Among CD4+ T cell, Follicular helper T cells (Tfh cells) are special one which are necessary for producing high affinity antibodies. Meanwhile Tfh cells can secrete IL21 and IL4 and show high expression of CXCR5, ICOS, PDCD1 (PD-1), and chemokine CXCL13, which also affect gastric cancer prognosis [10]. Cytotoxic CD8 T lymphocytes are present in tumors and their functions in recognizing tumor epitopes are nevertheless generally important in antitumor reaction [11]. And CD8 T cells are an important factor on the initial development of tumors, especially in existing tumor, and the presence of CD8 T cells indicates poor prognosis [12].

### 2.2. Regulatory T Cell

Regulatory T cells (Tregs) are a kind of T lymphocytes with an immunoregulatory capacity, which can inhibit the proliferation and cytokine secretion of effector T lymphocytes. Giving this function, inappropriate production or dysfunction of Tregs could result in severe damage of the host immune system [13]. In recent years, regulatory T cells (Tregs) within tumors, also known as tumor infiltrating Treg cells, have been considered to play a key role in immune evasion [13]. And Tregs are correlated with progression and poor outcomes in gastric cancer ([2]; [14]), but the relation between tumor infiltrating T cells and gastric cancer is unclear.

### 2.3. Others

In addition, many other related cells and molecules also play a role in prognosis of gastric cancer. Dendritic cells (DC) play the central role in cancer immunosurveillance as the antigen-presenting cells (APC) are involved in the antitumor immune responses [15]. T cell immunoglobulin and mucin domain-3 (Tim-3) is negative regulatory molecules and plays a major role in the tumor immunological tolerance [16]. And B7-H1 (also known as PDL1) is a member of the B7 superfamily [17]. PD-L1 expression has been detected in cancers of the skin [18], lung [19], breast [20], kidney [21], bladder [22], esophagus [23], stomach [24], head, and neck [25], among others. B7-H4 is a coinhibitory molecule which negatively regulates T cell immunity and is rarely expressed in resting antigen-presenting cells (APCs) [26] but is upregulated in a variety of cancer tissues including ovary, kidney, stomach, lung, and pancreas [27–29].

## 3. The Correlation between T Cells and Gastric Cancer

The factors that can affect progress and prognosis of the gastric cancer patients are various, such as TNM stages [30], invasive depth, lymph node metastasis ratio [31], and tumor immunity. And there are three molecular carcinogenesis mechanisms which may be correlated with GC: chromosomal instability [32], microsatellite instability (MSI) [33], and CpG island methylator phenotype [34]. T cell immunity also plays a role in these mechanisms, and some researches have already suggested that beta-catenin/T cell factor- (TCF-) mediated transcription (canonical Wnt signaling) could result in chromosomal instability (CIN) [35], and MSI gastric cancers possibly express more PD-L1 and have increasing CD8+ T cells before tumor invasive [36].

Nowadays, since the function of T cell immunity in cancer is researched more and more clearly, we found that it can also influence the progress and prognosis of GCs directly or indirectly participating in antitumor responses. For example, DC as the antigen-presenting cells (APC) is involved in the antitumor immune responses, while CD8+ T cell may dissolve and kill tumor cells, and CD4+ cell (including Foxp3+ Tregs) impose restrictions on tumor response.

Except these cells, many molecule such as Th17 [37], CD133 [38], gastrokine 1 [39], angiogenic factor [40], and LKB1 (Sun J et al., 2016) also have possible impact on diagnosis, progress, treatment, and prognosis of gastric cancer.

## 4. The Correlation and Mechanism between T Cells Immunity and Prognosis of Gastric Cancer

T cell immunity is important in antitumor response and studied in many other cancers. Some studies also show there could be correlation between gastric cancer prognosis and T cells. Haas et al. introduced a phenomenon in their experiment that an increasing stromal FoxP3+ TIL infiltration in tumor issues had a negative correlation with UICC- stage (Pearson's correlation coefficient, *r* = −0.40; *p* = 0.001), number of lymph node metastases (*r* = −0.36; *p* = 0.009), and N category in general (*r* = −0.36; *p* = 0.023). But this relationship could not be seen in other cell types [41]. Cheng et al. showed that Tim-3 was expressed in CD4+ T cells and CD8+ T cells higher in gastric issues and had a meaningful relation with tumor invasion and TNM stage, which could lead to poorer prognosis [16]. And Qing et al. had confirmed that PD-L1 could express more in highly differentiated gastric cancers, and it had an obvious relationship with the depth of invasion (odds ratio [OR] = 3.37; *p* = 0.005), lymph node metastasis (OR = 2.68; *p* = 0.020), tumor differentiation (OR = 3.19; *p* = 0.008), pathological type (*χ*^2^ = 8.676; *p* = 0.013), and survival time (OR = 3.39; *p* = 0.003) [42]. And they proposed that targeting the PD-L1 and APE1 signaling pathways may be a new treatment for gastric cancer, especially deep invasion and lymph node metastasis [42]. Cho et al. indicated that PD-L1 expression was frequently correlated with a lower risk of lymph node metastasis (*p* = 0.027) and lower tumor stages in intestinal type cancer by the Lauren classification [43]. But the mechanism between gastric cancer prognosis and T cells immunity is not very sure.

### 4.1. CD4+ T Cell and CD8+ T Cell

There are some researches focusing on the relationship between the subsets of CD4+ T cell and the progress and prognosis of gastric cancer. Shen et al. found that CD4+CD25+CD127low/− Tregs are correlated with more advanced stage of gastric cancer through suppressed effector T cell proliferation and express Foxp3 [44]. In another research, Kindlund et al. suggested that CD4+ regulatory T cells can promote tumor growth by inhibiting T cell mediated tumor cell killing, depending on IL-10 and/or TGF-*β*, but they also showed that CD4+CD25^High^ expresses higher IL-10 [2]. As we all know,* Helicobacter pylori* infection is related to prognosis of GCs. Zhang et al. investigated the potential functions of Follicular helper T cells in the GCs with* Helicobacter pylori* infection. His group found that Th1 and Th17 are the most common subsets of Follicular helper T cells and can be negatively correlated with the disease-free survival of tumor resection [45].

And CD8+ T cells have also been studied. Lu et al. indicated that GC patients with high-density CD8+ had higher overall survival rates than low-density ones by Kaplan-Meier test in MSI-high GCs [11]. But Thompson et al. demonstrated that tumors with high CD8+ T cell density either in intratumor or in stromal had worse progression-free survival (PFS) and OS compared with the lower ones [17]. Tuncel et al. introduced patients with lower numbers of CD8+ T lymphocytes in the tumor, which has a negative correlation with HLA-G and had a poorer prognosis [46].

### 4.2. Foxp3+ Treg Cell

 Although the functions of some immunity cells have been recognized by studies, some controversies are still present.

Foxp3+ Tregs are the most concerned cell and their function is still controversial. Hou et al. showed the level of FoxP3+ Tregs in gastric cancer tissues related to an advanced clinicopathological stage and lymph node metastasis, which indicted poor prognosis [13]. Yuan et al. found that the level of FoxP3 is higher in Tregs and it can inhibit the proliferation of autologous CD4+CD25−T cells in a COX-2-dependent manner to lead to poor prognosis which can be reversed by COX inhibitors [14]. In another article, Tuncel et al. got the similar conclusion that the high numbers of Tregs in the primary tumor, positive regulated by HLA-G, are associated with poor prognosis [46]. But there are some researches having opposite conclusions. Kim et al. showed that GCs with high-density FoxP3+ TILs had significantly higher overall survival rates and low density is closely related to a higher TNM stage, invasion depth, and lymphatic and vascular invasion and proved FoxP3+ T cell density in the intraepithelial cells was an independent predictor for overall survival. But the result of Kim et al. is confined to microsatellite-unstable gastric cancers [47]. Haas et al. suggested that high level of Treg is associated with improved outcome probably via inhibiting local inflammatory process [41]. And Feichtenbeiner et al. found an interesting conclusion that prognostic effect of TILs cells in gastric cancer depends on the distance within cells, and FoxP3+ TILs must be located within 30 and 110 *μ*m far from CD8+ T cells to play its positive impact on prognosis [48]. In addition, Ma et al. investigated the expression of FoxP3 protein in tumor cells and they showed that the high level predicts a good prognosis, whereas high-density Treg is opposite [49].

## 5. Other Cells and Molecule Related T Cell Immunity in GCs

Except mainly cells in T cell immunity, there are some other cells and molecule also could have their functions in GCs.

Kashimura et al. suggested that the density of CD83+ DCs in negative lymph nodes was an independent prognostic factor by multivariate analysis for patients with metastatic lymph nodes [9]. Gao et al. descripted that the overexpression of B7-H1 in carcinomas has been shown to induce apoptosis in the effector T cells to repress T cell activation and proliferation, which led to lower 5-year OS and DFS [50]. Geng et al. confirmed that lymph node metastasis and B7-H1 overexpression were independent prognostic factors which are negative with gastric cancer through Cox regression multivariate analysis [51]. Shi et al. proposed that soluble B7-H4 (sB7-H4) in circulation is a valuable molecule for predicting the progression and prognosis of GCs and a positive correlation between the two things [52]. And Chen et al. suggested that the expression of T-bet, a key marker for type 1 immune responses, can serve as a prognostic indicator which has negative effect [53]. In a recent study, Kim et al. revealed that decreasing NOVA1 expression in tumor tissue was related to tumor progression and poor prognosis via immune dysfunction of T cells and macrophages [54]. And Th12 [55], Th17 [37, 56], Th1 [57], CX3C chemokines [58], diversity index of mucosal resident T lymphocyte [59], myeloid derived suppressing cells [60], immune activating receptor NKG2D [61], CCR7 [62], and IL-10 [63] are also involved in the progression and prognosis of GCs.

Another interesting thing is the prognosis and progress of the same immunity cells can vary under different locations. Tim-3 is a negative regulatory molecule, only when it overexpresses CD8+ T cell or Tregs can lead to poor prognosis. Another protein is Foxp3+, its higher expression in tumor cells predicts good outcome but in Tregs the function is inverse. It has different prognosis when acting on different cells. Cheng et al. found that CD4+ and CD8+ T cell can be upregulation in GCs by Tim-3, but CD4+ T cell has poorer prognosis. Tim was also an independent factor for GCs, and the lower is the better [16]. Shen et al. revealed that the level of Tim-3 is up in both* H*.* pylori*-infected asymptomatic and gastric cancer patients, which is on Tregs and CD8+ T cells associated with worse prognosis [64]. And Milašienė et al. observed that higher levels of the absolute number of lymphocyte had a positive effect on overall survival of gastrium in stage III, but there is no effect in stage II [65].

## 6. Forecast

The subsets of immunity cells have their own special role in response to gastric cancer and lead to different outcomes of patients. But the function is affected by location, category, related molecule, interaction between the cells, and so on. Definite function is still unclear and needs more studies. More studies are needed to investigate the relationship between the T immunity cell and gastric cancer, especially forcing on Foxp3+ Tregs and the influence of location and mutual relations between cells. At the same time, I think that the role of memory T cell is ignored in the progress and prognosis of GCs, and more research is essential.

Because of the T cells exhibiting a possible relationship in the progress and prognosis of GCs, it may provide new theory and way on diagnosis and treatment of gastric cancer. More studies are needed.
